# Viral mimicry evasion: a new role for oncogenic KRAS mutations

**DOI:** 10.1002/1878-0261.13771

**Published:** 2024-11-26

**Authors:** Raymond Chen, Aobo He, Daniel D. De Carvalho

**Affiliations:** ^1^ Department of Medical Biophysics University of Toronto Canada; ^2^ Princess Margaret Cancer Centre University Health Network Toronto Canada

**Keywords:** colorectal cancer, DDX60, dsRNA, immune checkpoint inhibition, KRAS, viral mimicry

## Abstract

“Viral mimicry” refers to the induction of an innate immune response and interferon signaling by endogenous stimuli such as double‐stranded RNA (dsRNA). This response has been shown to have strong cancer therapeutic potential, including by enhancing the effectiveness of immune checkpoint inhibition (ICI) therapies, and may represent a tumor suppression mechanism that needs to be overcome for malignant transformation to proceed. In a recent study, Zhou and colleagues identify KRAS, a frequently mutated oncogene, as a negative regulator of dsRNA and viral mimicry in an ICI‐resistant colorectal cancer model. Oncogenic KRAS^G12D^ mutations downregulate the RNA‐binding protein DDX60 by activating the AKT signaling pathway, which inhibits STAT3, a critical transcription factor regulating DDX60 and other interferon‐stimulated genes. Overexpression of DDX60, which competitively binds to dsRNA to prevent RISC‐mediated degradation, or targeting of KRAS^G12D^ elevated dsRNA levels, resulting in viral mimicry activation and potentiation of ICI treatment. These results establish KRAS as a promising target to sensitize immune “cold” tumors to ICI therapy and demonstrate the potential role of oncogenic mutations in viral mimicry evasion during tumorigenesis.

## Graphical abstract

“Viral mimicry” is a potent anticancer mechanism involving innate immune activation by endogenous nucleic acids like double‐stranded RNA (dsRNA). Zhou and colleagues report viral mimicry evasion as a novel function of oncogenic KRAS mutations by suppressing the RNA helicase DDX60, leading to poor immune checkpoint therapy responses. Their study highlights the KRAS‐DDX60 axis as a promising target to sensitize immune “cold” tumors.

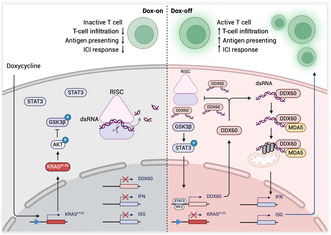

**Abbreviations**
CRC: colorectal carcinoma
Dox: doxycycline
dsRNA: double‐stranded RNA
ICI: immune checkpoint inhibition
IFN: interferon
iKAP: Dox‐inducible *Kras* ^*G12D*^ and *Apc* and *Trp53* null mutation murine model
IR‐Alu: inverted repeat‐Alu
ISG: interferon stimulated gene
LUAD: lung adenocarcinoma
MSI‐H: microsatellite instability‐high
MSS: microsatellite stable
PDAC: pancreatic ductal adenocarcinoma
poly(I:C): polyinosinic:polycytidylic acid
TE: transposable elements

Activation of transposable elements (TEs) can lead to dsRNA formation and stimulation of pattern recognition receptors such as RIG‐I or MDA5, triggering an interferon (IFN) response—a process termed “viral mimicry” [1, 2]. TE‐mediated dsRNA formation frequently occurs at young inverted repeat Alu elements (IR‐Alus) located within the same RNA molecule, allowing formation of a hairpin [3]. A growing body of evidence shows that the viral mimicry response has strong antitumor effects, including loss of cancer cell fitness and activation of antitumor adaptive immune responses [4]. In line with these antitumor effects, several tumor suppressor genes, such as TP53 and RB [5], and several anticancer therapies were shown to induce viral mimicry responses as one of their mechanisms of action [4]. Furthermore, viral mimicry responses are often counterbalanced during malignant transformation. For instance, some cancer cells become dependent on adenosine‐to‐inosine (A‐to‐I) editing of IR‐Alus by the deaminase ADAR1, compromising the double‐stranded structure of Alu elements [3], while other cancer cells become dependent on IR‐Alu dsRNA decay by the exonuclease XRN1 [6, 7]. Altogether, these observations suggest a “fire alarm hypothesis” where viral mimicry induced by TEs can act as a surveillance system for dysregulated homeostasis that culls precancerous cells [8]. This fire alarm hypothesis suggests that viral mimicry is a central and underappreciated tumor suppressor mechanism that needs to be overcome in order for malignant transformation to thrive [8]. However, to the best of our knowledge, there was no clear evidence that oncogenic mutations could directly cause viral mimicry evasion yet. In a recent paper, Zhou and colleagues [9] link activating KRAS mutations with suppression of dsRNA accumulation and viral mimicry via the AKT‐STAT3‐DDX60 axis. This provides both a promising avenue to sensitize immune “cold” tumors to immune checkpoint inhibition (ICI) therapy and an important conceptual advancement that oncogenic mutations may promote transformation by actively circumventing the tumor suppressive viral mimicry alarm.

KRAS is among the most frequently mutated genes in cancer patients, with oncogenic mutations found in ~ 30% of lung adenocarcinomas (LUAD), ~ 95% of pancreatic ductal adenocarcinomas (PDAC), and ~ 45% of colorectal carcinomas (CRC) [10]. Activating KRAS mutations, which stimulate the MAPK and AKT signaling pathways to promote cell proliferation and survival, have been associated with immune evasion and resistance to ICI therapy in CRC [11, 12]. Moreover, the majority of CRC patients harboring KRAS mutations are classified as the immune “cold” microsatellite stable (MSS) subtype. This subtype displays a poorer immune infiltration and response rate to ICI therapy compared with the microsatellite instability‐high (MSI‐H) subtype [13], suggesting KRAS inhibition as a potential strategy to convert “cold” tumors to “hot” ones. While KRAS mutations, which occur predominantly at codons 12 (G12) and 13 (G13) in CRC, were previously thought to be undruggable, recent efforts have led to the development of several small‐molecule inhibitors such as the G12C inhibitors sotorasib and adagrasib as well as the G12D inhibitor MTRX1133 [14].

In a recent paper, Zhou et al. [9] studied the effect of KRAS on viral mimicry utilizing a murine CRC model that is doxycycline (Dox)‐inducible for the *KRAS* ^*G12D*^ mutant and null alleles of *Apc* and *Trp53* (iKAP) (Fig. 1). Previously, the authors found that oncogenic KRAS suppresses IRF2‐mediated interferon signaling in this model, resulting in an immunosuppressive tumor microenvironment and resistance to ICI therapies [11]. In this study, they establish dsRNA as the critical mechanistic link between KRAS mutation and IFN signaling. Extinguishing the KRAS^G12D^ mutation by removing doxycycline (dox‐off) from the iKAP cells increased dsRNA levels, including those derived from repetitive elements and the mitochondria, and upregulated interferon‐stimulated genes (ISGs) in a RIG‐I‐ and MDA5‐dependent manner (Fig. 1). Additionally, both KRAS^G12D^ mutant cells and wild‐type cells with KRAS^G12D^ overexpressed showed blunted responses to exogenous treatment with the dsRNA analogue polyinosinic:polycytidylic acid (poly(I:C)).

**Fig. 1:**
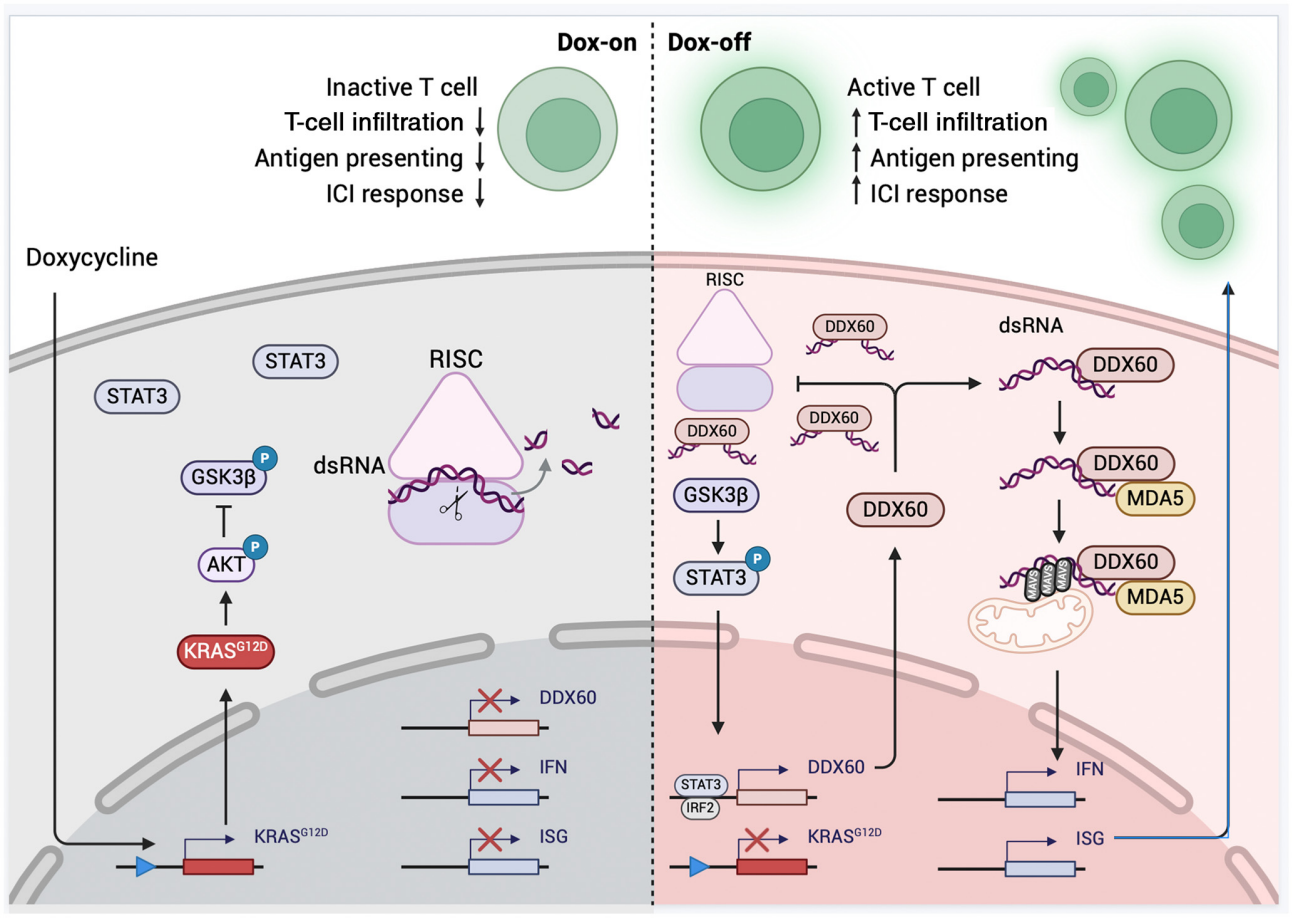
Oncogenic KRAS^G12D^ mutation destabilizes cytosolic dsRNA via the AKT‐STAT3‐DDX60 axis. (Left) Doxycycline treatment (dox‐on) induces the KRAS^G12D^ mutation, which phosphorylates AKT and inhibits GSK3β via phosphorylation. This prevents activation of STAT3 from triggering DDX60 expression. Without DDX60, cytosolic dsRNAs are degraded by the RISC complex, resulting in a lack of IFN response and an immune “cold” phenotype that is resistant to ICI treatment. (Right) When doxycycline is removed (dox‐off), oncogenic KRAS expression is suppressed and GSK3β can phosphorylate STAT3, which enters the nucleus and promotes DDX60 expression. DDX60 displaces RISC by competitively binding dsRNAs, preventing their degradation. The subsequent accumulation of cytosolic dsRNA is detected by pattern recognition receptors like MDA5 and RIG‐I, triggering viral mimicry and downstream IFN responses that produce an ICI‐sensitive immune “hot” phenotype by promoting T‐cell infiltration and activation.

The authors go on to attribute the effect of KRAS^G12D^ on dsRNA levels to DDX60, an IFN‐inducible RNA helicase identified through RNA‐seq data as downregulated in KRAS^G12D^‐mutated cells compared with KRAS wild‐type. *KRAS* ^*G12D*^ overexpression and knockdown experiments confirmed the negative correlation between mutation status and DDX60 levels. Mechanistically, KRAS/AKT signaling suppresses the JAK–STAT pathway by preventing GSK3β‐mediated activation of STAT3, a critical transcription factor in IFN signaling that also upregulates DDX60. Once expressed, DDX60 appears to enhance stability by competitively binding to dsRNA to prevent RISC‐mediated degradation. Accordingly, DDX60 overexpression and subsequent increases in dsRNA levels resulted in IFN activation, elevated antigen presentation, and increased CD8^+^ T‐cell infiltration [9]. In accordance with their previous results [11], the authors also found that DDX60 upregulated IRF2, which in turn promoted further DDX60 expression in a positive feedback loop. Critically, an intact immune system is required as these effects were accompanied by a concomitant decrease in MC38K tumor growth only in C57BL/6J mice and not nude mice. The authors also observed that DDX60 overexpression synergized with ICI treatment in both *in vivo* mouse models and an *in vitro* human coculture system of primary peripheral blood mononuclear cells as well as the HCT116 (MSI‐H) and SW620 (MSS) cell lines.

These results hold high translational promise, but it will be critical to replicate these findings in a more clinically relevant context such as patient‐derived samples. Similarly, while the authors only tested dsRNA levels *in vitro* with MRTX1133, it would be of clinical importance to see whether the other results can be achieved by a small molecule inhibitor as well. It would also be of interest to see whether these findings extend to other KRAS mutants such as KRAS^G12C^, which can also be targeted to produce an antitumor immune response [15], or to other KRAS‐mutated cancer contexts such as lung adenocarcinoma.

Overall, Zhou et al. demonstrate that targeting the KRAS/DDX60 axis is a novel and effective method to elevate intracellular dsRNA levels, thereby activating viral mimicry and potentially converting immune “cold” tumors to immune “hot” ones. In addition to clinical relevance, these findings also highlight the mechanistic importance of post‐transcriptional dsRNA regulation as a modulator of viral mimicry responses. In particular, this work complements previous studies showing that LSD1 inhibition can induce interferon responses in part by downregulating RISC components [16] and that DHX9, another RNA‐binding helicase, can inhibit viral mimicry [17]. Finally, these results represent early evidence suggesting that oncogenic mutations themselves can directly lead to cytosolic dsRNA depletion as a strategy to not only evade the immune system in a therapeutic context (e.g. ICI resistance) but potentially also to bypass culling by the viral mimicry alarm as an early transformation event.

## Conflict of interest

DDDC reports support from Adela, Inc outside the submitted work. The other authors declare no conflicts of interest.

## Author contributions

DDDC and RC conceived the structure and the outline of the article; RC and AH wrote the original draft of the article; DDDC reviewed and revised the original draft and the figure. All authors have reviewed and agreed to the published version of this article.
